# Postoperative contralateral renal rupture with multisystem complications after surgery for right renal calculi: a rare clinical case report

**DOI:** 10.1186/s12893-026-03773-8

**Published:** 2026-04-24

**Authors:** Yonglin Huang, Jian Yang, Ke Zeng, Jing Zou

**Affiliations:** 1 Department of Urology, Rongxian People’s Hospital, Zigong, Sichuan 643100 China; 2 Department of Urology, Zigong First People’s Hospital, No. 42 Shangyi Haoyi 1st Branch Road, Ziliujing District, Zigong, Sichuan 643000 China

**Keywords:** Spontaneous renal rupture, Wunderlich syndrome, Multisystem complications, Multidisciplinary team (MDT)

## Abstract

**Background:**

Spontaneous renal rupture (Wunderlich syndrome) is a rare and life-threatening condition, typically occurring in kidneys with pre-existing ipsilateral pathology. The occurrence of spontaneous contralateral renal rupture with multisystem complications shortly after urological surgery is extremely uncommon. This case report describes the diagnosis and treatment of contralateral renal rupture following right renal calculi surgery, complicated by infection, acute respiratory distress syndrome (ARDS), and chylothorax. It also discusses the importance of evaluating subtle contralateral renal abnormalities and employing multidisciplinary team (MDT) management.

**Case presentation:**

A 52-year-old male patient was admitted for right renal calculi. Preoperative non-contrast CT revealed a mild, atypical hypodense lesion in the left kidney, which was not further characterized preoperatively. On the second day of hospitalization, the patient underwent right kidney surgery. Three days postoperatively, he suddenly developed left flank pain, hypotension, and a drop in hemoglobin levels. Contrast-enhanced CT confirmed spontaneous rupture and massive hemorrhage of the left kidney. Digital subtraction angiography revealed arterial extravasation, and selective embolization was performed. The patient was subsequently transferred to the ICU. He later developed ARDS, co-infection with influenza virus and bacterial/fungal pathogens, and left-sided chylothorax. Through comprehensive treatment including mechanical ventilation, antimicrobial therapy, thoracic drainage, and nutritional support, and under the coordination of the MDT, the patient’s condition gradually improved, and he was eventually discharged after full recovery.

**Conclusion:**

Even mild or atypical hypodense lesions in the contralateral kidney may indicate potential bleeding risk and should prompt further evaluation with enhanced CT or MRI prior to surgery. Postoperative stress response, inflammation, and hemodynamic fluctuations may trigger rupture of vulnerable pre-existing renal lesions. In patients presenting with flank pain, anemia, and shock after surgery, spontaneous rupture of the contralateral kidney should be highly suspected, and prompt imaging and interventional management are critical. In the presence of multisystem complications such as ARDS, mixed infections, and chylothorax, MDT management is vital for improving patient outcomes.

**Supplementary Information:**

The online version contains supplementary material available at 10.1186/s12893-026-03773-8.

## Introduction

Spontaneous renal rupture, also known as Wunderlich syndrome, is a rare but potentially life-threatening urological emergency characterized by atraumatic subcapsular or perirenal hematoma formation [1]. Its etiologies include benign and malignant renal tumors—particularly angiomyolipoma—cystic lesions, infections, vascular malformations, stone-related obstruction, anticoagulation therapy, and coagulation disorders [2, 3]. Clinically, it often presents with acute flank pain, hypotension, and even hemorrhagic shock, and delayed recognition can rapidly endanger the patient’s life [4].

Although spontaneous renal rupture is uncommon, it typically occurs in kidney with a known underlying lesion [5], whereas rupture of the contralateral, non-operated kidney is exceedingly rare. Previous reports indicate that such events are frequently associated with subtle or previously uncharacterized renal parenchymal abnormalities, such as small fat-containing lesions, cystic changes, or focal vascular anomalies [6]. However, when these lesions appear only as mild and nonspecific low-density areas on preoperative non-contrast CT, they are easily overlooked, making risk assessment challenging. It is noteworthy that major surgery induces a significant physiological stress response—including activation of the sympathetic–adrenal system, increased inflammatory mediators, hemodynamic fluctuations, and transient imbalance in coagulation and fibrinolysis—all well documented in the postoperative period [7–9]. These systemic alterations may compromise the structural stability of otherwise silent or fragile renal lesions, potentially precipitating bleeding under certain conditions.

The uniqueness of this case lies not only in the occurrence of contralateral spontaneous renal rupture but also in the development of multiple severe postoperative complications, including bilateral pneumonia, acute respiratory distress syndrome (ARDS), influenza A/B viral infection, urinary fungal infection, chylothorax, and profound hemodynamic instability. The involvement of multiple organ systems led to rapid clinical deterioration and greatly increased the complexity of management, underscoring the essential role of a multidisciplinary team (MDT) in treating critically ill patients. Reports of postoperative urological cases with such extensive multisystem complications remain extremely rare in the literature. Against this background, the present case holds significant clinical relevance: (1) it emphasizes the importance of careful bilateral renal imaging assessment before urological surgery, even when only mild low-density changes are present; (2) it highlights the possible contribution of postoperative stress, inflammation, and coagulation shifts to the rupture of pre-existing renal lesions; (3) it analyzes the pathophysiology and management challenges of multisystem complications; and (4) it reinforces the value of MDT collaboration in managing complex, critically ill patients. For these reasons, we report a rare case of contralateral spontaneous renal rupture following right renal surgery, complicated by severe infection, ARDS, and chylothorax, to provide additional clinical insights. This case report adheres to the SCARE 2020 guidelines for surgical case reports [10].

## Case presentation

**Fig. 1:**
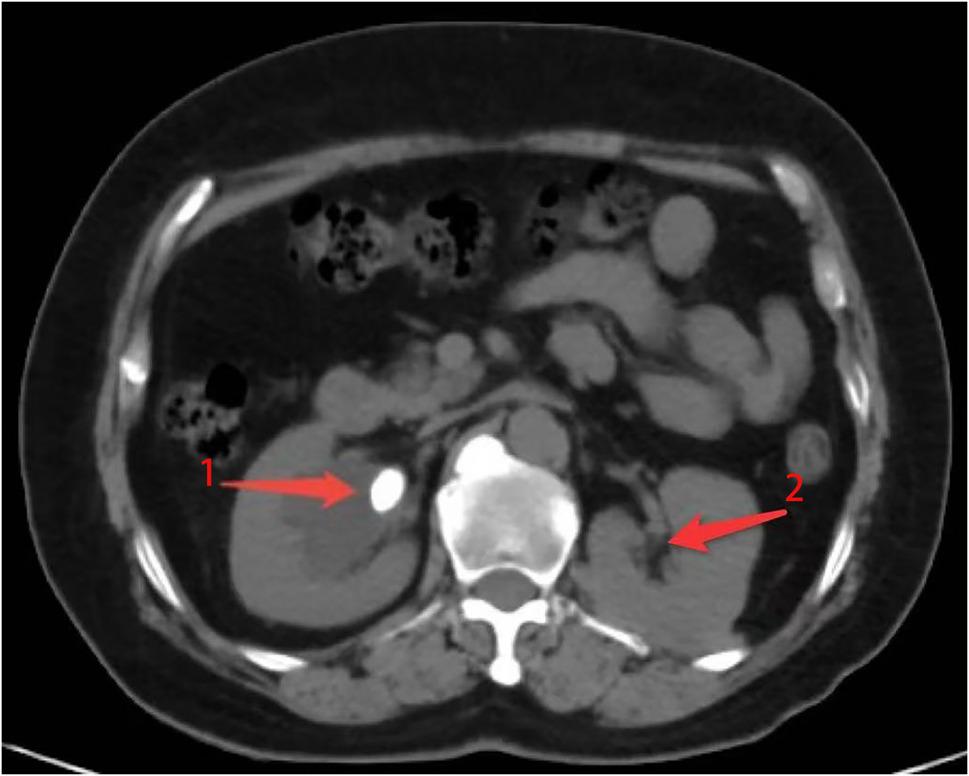
Abdominal CT scan showing findings in the right and left kidneys. Arrow 1 indicates a hyperdense right renal calculus with mild hydronephrosis, measuring approximately 2.1 × 1.4 cm. Arrow 2 points to mild, atypical hypodense lesions in the left kidney

The patient, a 52-year-old male, had no significant past medical history or chronic illnesses. He presented to a local hospital with recurrent right flank pain accompanied by urinary irritation symptoms. A preoperative non-contrast abdominal CT scan revealed high-density calculi within the right renal collecting system, along with mild dilation of the renal pelvis and calyces. The left kidney appeared morphologically irregular, with patchy and punctate hypodense lesions (up to 1.3 cm in size), some of which showed fat density. There was no perirenal fluid collection, and no hyperdense lesions within the renal parenchyma (Fig. 1). The patient had no left flank pain, no hematuria, normal body temperature, and normal renal function, with no signs of infection. The patient had no left flank pain, hematuria, fever, or renal function impairment. Although the presence of fat density raised the possibility of an angiomyolipoma, the lesion was not formally reported as such by the radiologist, likely due to its atypical patchy morphology, small size, and the absence of contrast-enhanced imaging. Given that the patient’s symptoms were entirely localized to the right kidney, these left-sided imaging findings did not prompt further preoperative evaluation with contrast-enhanced CT or MRI.

**Fig. 2:**
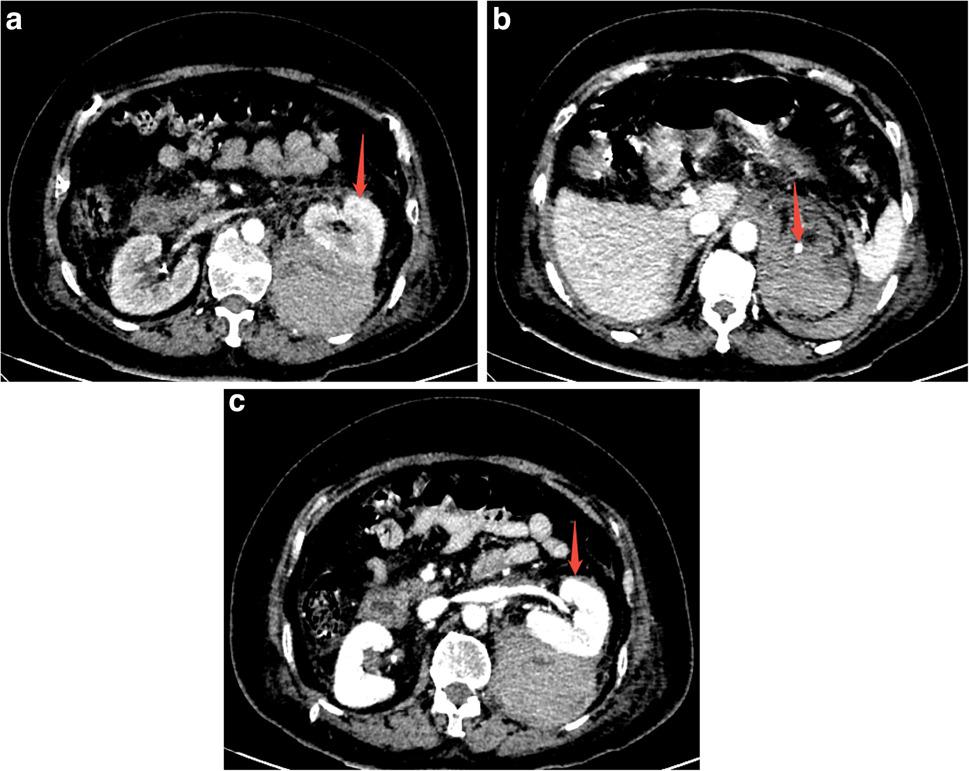
**a** Contrast-enhanced abdominal CT scan after hemorrhage. Arterial-phase enhancement is observed in the left kidney, indicated by the red arrow, suggesting an active renal parenchymal lesion following the bleeding episode. **b** Venous-phase contrast-enhanced CT scan after hemorrhage. The venous-phase image shows a focal area of persistent enhancement in the left kidney (red arrow), corresponding to the suspected bleeding site. **c** Venous-phase contrast-enhanced CT scan after hemorrhage. A venous-phase image demonstrates a persistently enhanced focus in the left kidney (red arrow), consistent with an active or recent bleeding site

**Fig. 3:**
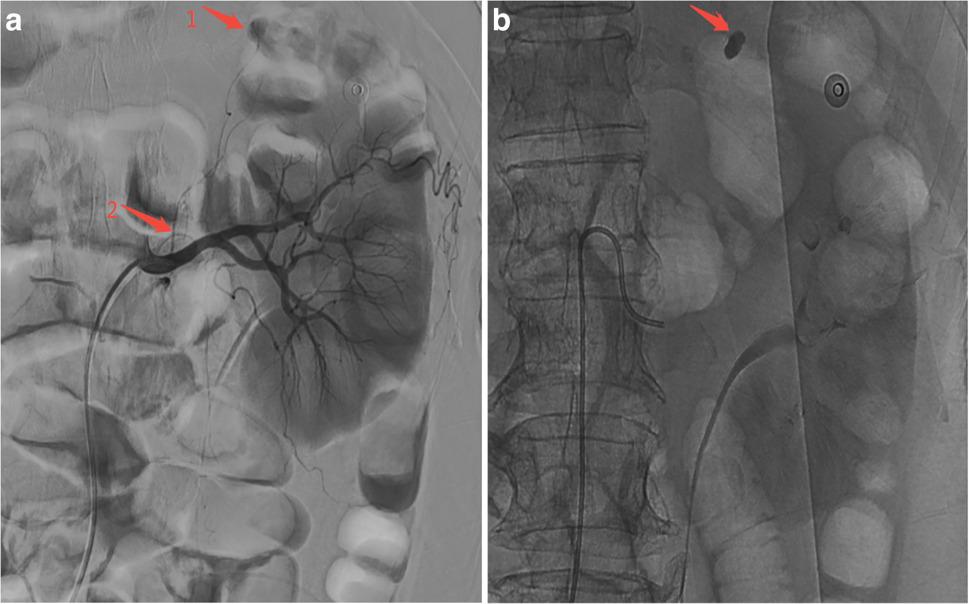
**a** DSA of the left renal artery. Marker 1 indicates the bleeding point in the upper pole of the left kidney, and marker 2 shows the arterial branch supplying the upper pole. **b** Post-embolization angiographic image. The red arrow indicates residual contrast medium retention after coil embolization

On the second day after admission, the patient underwent a series of right-sided urological procedures under general anesthesia. The surgeries included: percutaneous nephrolithotomy (PCNL) with laser lithotripsy, transurethral ureteral lesion laser resection, transurethral ureteral catheterization, transurethral ureteral stent insertion, and percutaneous nephrostomy. The total operative time was 65 min. The estimated blood loss was minimal, and no blood transfusion was required. Anesthesia records showed that the patient remained hemodynamically stable throughout the procedure, with systolic blood pressure consistently maintained without the need for vasopressor support. The procedure proceeded without complications. However, at 10:00 PM on the same day, he suddenly developed nausea and vomiting, which was immediately followed by the onset of acute left flank pain. The pain was managed with symptomatic treatment and temporarily subsided. On postoperative day 1, he developed a low-grade fever, fatigue, and mild cough, but remained hemodynamically stable. On postoperative day 2, the fever worsened (up to 38.5 °C), accompanied by chills, chest tightness, and progressively worsening fatigue. Complete blood count revealed an increased neutrophil percentage, and C-reactive protein (CRP) was markedly elevated, indicating a postoperative infection. Auscultation revealed scattered moist rales in both lungs, along with mild dyspnea. In the early hours of postoperative day 3, the patient suddenly experienced severe pain in the left flank and abdomen, accompanied by pallor, cold sweats, and marked fatigue. His blood pressure dropped to 85/60 mmHg, and hemoglobin fell to 75 g/L. Emergency contrast-enhanced abdominal CT revealed discontinuity of the left renal capsule and a large perirenal hematoma of mixed density, with areas of high attenuation suggestive of active arterial bleeding. In addition, perirenal fascial thickening and anterior displacement of the renal fascia were noted, leading to a definitive diagnosis of spontaneous rupture of the left kidney with massive hemorrhage (Fig. 2a, b and c). Emergency management protocols were immediately initiated. The patient was transferred to the interventional radiology department for left renal artery angiography. Digital subtraction angiography (DSA) showed active contrast extravasation from a small branch at the upper pole of the left kidney, confirming arterial rupture as the source of bleeding. However, no cystic structures or signs of a pseudoaneurysm were observed (Fig. 3a and b). Upon retrospective image review, the 1.3 cm fat-containing hypodense lesion identified on preoperative non-contrast CT was precisely located in the upper pole of the left kidney and was supplied by the same upper pole arterial branch from which the bleeding originated. The interventional team proceeded with selective arterial embolization, after which the patient’s hemodynamics gradually stabilized. 

Within a few hours after the embolization procedure, the patient developed significantly worsening dyspnea, with oxygen saturation dropping below 85%. High-flow oxygen therapy was ineffective, and the patient was urgently transferred to the Intensive Care Unit (ICU) on postoperative day 4. During the resuscitation for hemorrhagic shock, the patient received 4 units of packed red blood cells and 200 mL of fresh frozen plasma. No platelets were transfused. The onset of hypoxemia and radiographic changes occurred 12 hours after the completion of transfusion. Chest CT revealed diffuse ground-glass opacities and focal consolidations in both lungs, along with interstitial thickening—findings consistent with ARDS. Arterial blood gas analysis indicated severe hypoxemia. The patient underwent endotracheal intubation and was placed on mechanical ventilation using a lung-protective ventilation strategy, along with appropriate sedation and analgesia. Upon transfer to the ICU, empiric broad-spectrum antibiotic therapy was immediately initiated with piperacillin-tazobactam (4.5 g every 8 hours), and continued based on clinical response. At the same time, an extensive infectious workup was initiated, and antimicrobial therapy was adjusted multiple times according to the results. On ICU day 2, respiratory pathogen antibody testing showed positive results for both influenza A virus IgM and influenza B virus IgM. Consequently, oseltamivir (75 mg orally twice daily) was started for antiviral therapy. On ICU day 3, urinalysis revealed a high yeast count (6,804.0/μL), and urine culture subsequently confirmed the growth of Candida glabrata, which was sent for susceptibility testing. Based on the preliminary findings, empiric antifungal therapy with fluconazole (400 mg loading dose, followed by 200 mg intravenously once daily) was started. On ICU day 4, left thoracic drainage produced 800 mL of chylous fluid. Pleural fluid analysis showed: triglycerides 120 mg/dL, total protein 3.4 g/dL, lactate dehydrogenase (LDH) 189 IU/L, nucleated cell count 2,780.0 × 10⁶/L, with lymphocyte predominance (62.2%). Bacterial culture of the pleural fluid was sterile. These findings confirmed the diagnosis of chylothorax. To exclude thoracic duct injury or central venous catheter-related trauma, bedside ultrasound was performed, which confirmed no signs of central venous catheter-related injury or thoracic duct rupture. The patient was managed with closed thoracic drainage, fasting, and total parenteral nutrition, followed by enteral nutrition with a low-fat, medium-chain triglyceride-based diet [11–13]. The drainage volume gradually decreased. On ICU day 5, blood cultures obtained on multiple occasions remained negative. Metagenomic next-generation sequencing of the bronchoalveolar lavage fluid also showed no pathogenic microorganisms. On ICU day 6, antimicrobial susceptibility testing for Candida glabrata, based on the urine culture from ICU day 3, showed intermediate susceptibility to fluconazole and sensitivity to voriconazole. On ICU day 7, after a clinical pharmacist consultation, fluconazole was discontinued and the regimen was changed to oral flucytosine (25 mg/kg every 6 hours) combined with intravenous caspofungin (70 mg loading dose followed by 50 mg once daily).

**Fig. 4:**
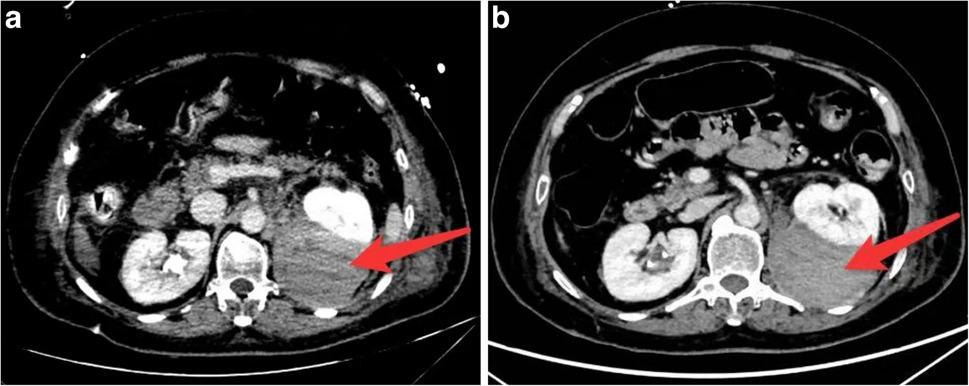
**a** CT image obtained on the first day after interventional surgery (hospital day 6), demonstrating a perirenal hematoma (red arrow). **b** CT image obtained on the 7th day after interventional surgery (hospital day 12), demonstrating a perirenal hematoma (red arrow)

**Fig. 5:**
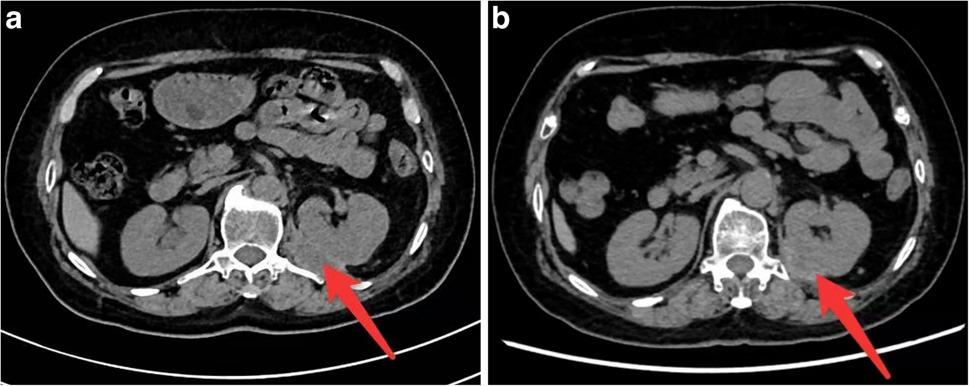
**a** CT image obtained on the day before discharge (hospital day 30) demonstrates a perirenal hematoma (red arrow). **b** CT image obtained 30 days after discharge demonstrates resolution of the perirenal hematoma (red arrow)

**Fig. 6:**
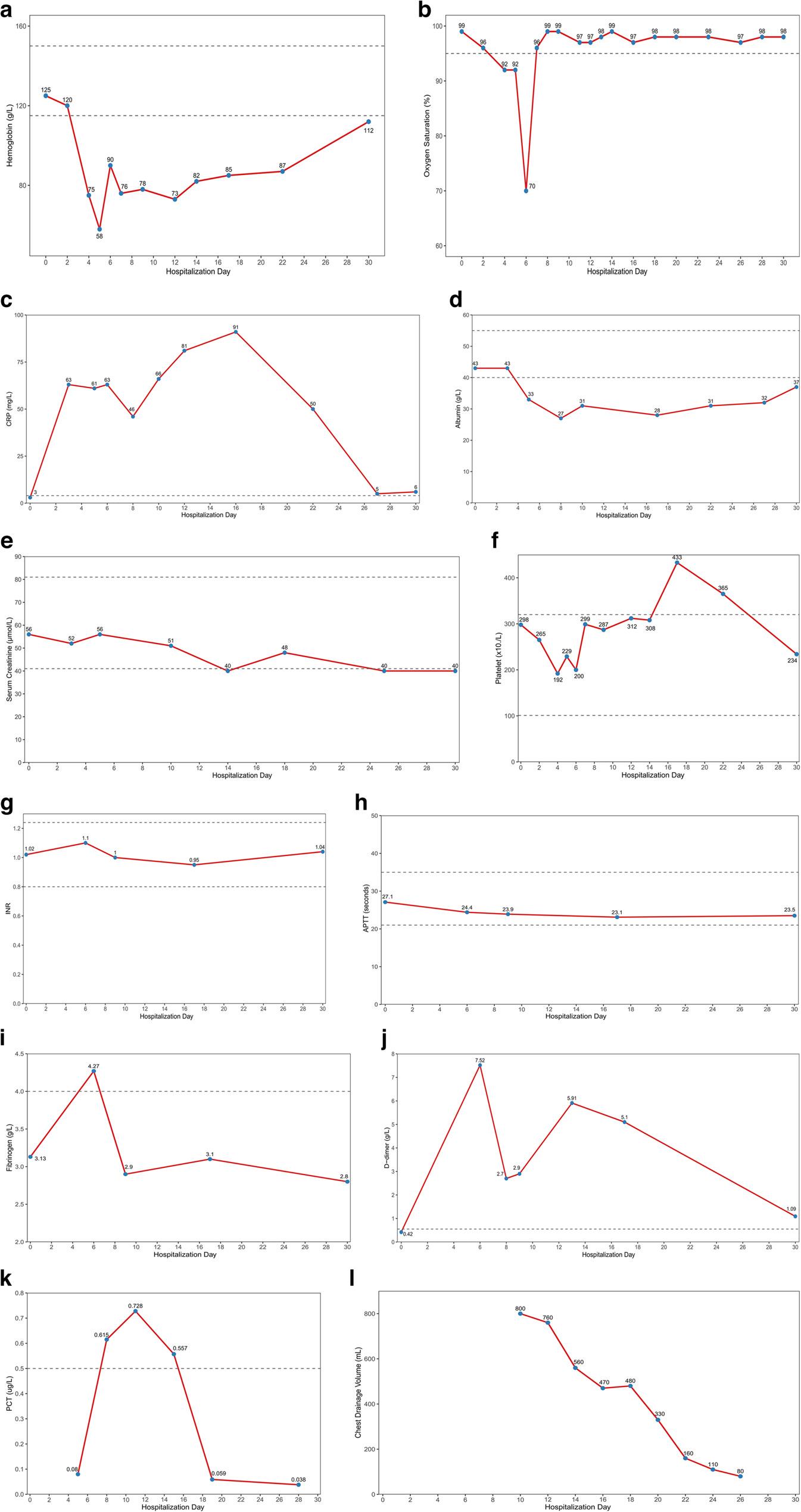
Serial clinical and laboratory findings during hospitalization (Day 0 = admission, Day 2 = surgery, Day 5 = interventional procedure, Days 6–22 = ICU stay). **a** Hemoglobin, (**b**) Oxygen saturation, (**c**) C-reactive protein (CRP), (**d**) Albumin, (**e**) Serum creatinine, (**f**) Platelet count, (**g**) International normalized ratio (INR), (**h**) Activated partial thromboplastin time (aPTT), (**i**) Fibrinogen, (**j**) D-dimer, (**k**) Procalcitonin (PCT), (**l**) Daily chest drainage volume. The dashed lines indicate the normal reference ranges for each parameter

During the 16-day ICU course, the patient required ongoing management for multiple organ dysfunctions, including the large perirenal hematoma secondary to left renal rupture, ARDS requiring mechanical ventilation, mixed viral-bacterial-fungal infection, and significant hypoproteinemia. A multidisciplinary team comprising urology, critical care, thoracic surgery, infectious diseases, and nutrition departments collaborated on the patient's comprehensive care. After treatment, chest imaging showed improvement in pulmonary inflammation, and the patient was successfully weaned off mechanical ventilation. The left pleural drainage output continued to decrease, allowing for chest tube removal. Inflammatory markers declined, and serial CT imaging demonstrated gradual resolution of the perirenal hematoma (Figs. 4a, b and 5a, b). No drainage was performed, as the patient was hemodynamically stable after embolization with no ongoing bleeding and lacked clinical or imaging signs of infection or mass effect, supporting conservative management. The patient was then transferred to the general ward for continued rehabilitation and subsequently discharged in good condition (Fig. 5a and b). Serial laboratory findings throughout hospitalization, including hemoglobin, oxygen saturation, CRP, albumin, serum creatinine, platelet count, international normalized ratio (INR), activated partial thromboplastin time (aPTT), fibrinogen, D-dimer, and procalcitonin (PCT), are summarized in Fig. 6a–k. The corresponding trend in daily chest drainage volume is presented in Fig. 6l. The peak and nadir values of these indicators are also summarized in Supplementary Table 1.

## Discussion

The key feature of this case lies in the spontaneous rupture of the contralateral kidney occurring shortly after surgery for right renal calculi, followed by a cascade of multisystem complications including arterial hemorrhage, ARDS, viral and fungal co-infections, and chylothorax. These pathological processes were interrelated and mutually reinforcing, resulting in rapid clinical deterioration and significantly increasing the complexity of management. The progression of the patient’s condition raises several important clinical questions worthy of in-depth consideration.

First, this case highlights the critical importance—and inherent challenges—of preoperative assessment of pre-existing renal lesions in the contralateral kidney. Although non-contrast CT prior to surgery had already revealed patchy and punctate hypodense lesions in the left kidney, some with fat density, these findings—despite the absence of contrast-enhanced imaging—should have been considered potential risk signals. However, due to the patient’s symptoms being localized to the right kidney, the abnormality did not receive sufficient attention and was not further evaluated. This ultimately led to a catastrophic rupture of the contralateral kidney postoperatively. Previous studies have noted that small angiomyolipomas with very low fat content, as well as certain fibrous-vascular lesions, often appear as nonspecific hypodense areas on non-contrast or even routine contrast-enhanced CT scans, and are therefore easily overlooked [14, 15]. Such pre-existing renal lesions may become unstable and rupture suddenly under specific stress conditions [16]. In this case, although the left renal lesion was small, it may have provided the pathological substrate for rupture. While the precise trigger for rupture remains uncertain, the temporal association with surgery raises the possibility that postoperative physiological changes may have played a contributing role. This underscores the need for heightened vigilance when encountering atypical or mildly abnormal findings on preoperative imaging, even if they seem clinically insignificant at the time. We acknowledge the lack of histopathological confirmation of the left renal lesion as a limitation. No contrast-enhanced imaging was performed preoperatively due to the patient’s asymptomatic status, and post-rupture biopsy was not feasible due to critical illness. Nevertheless, this limitation itself reinforces the core message: even mild, atypical fat-density findings on non-contrast CT warrant further evaluation, as they may harbor significant bleeding potential.

Secondly, this case offers clinical insight into a possible association between postoperative systemic stress responses in precipitating the rupture of pre-existing renal lesions. The rupture occurred in the early postoperative period, when the patient was under significant physiological stress. Extensive research has shown that surgery can activate the sympathetic nervous system, trigger the release of large quantities of inflammatory mediators (such as cytokines), cause hemodynamic instability, and disrupt the balance of the coagulation–fibrinolysis system [17, 18]. However, these mechanisms reflect general physiological responses to surgery and have not been directly implicated in spontaneous renal rupture. Theoretically, these systemic changes could contribute to an increased risk of renal hemorrhage through several mechanisms: (1) marked blood pressure fluctuations might generate shearing forces that compromise the integrity of fragile vessels within pre-existing lesions [19]; (2) inflammatory mediators could impair endothelial function and vascular wall stability [20, 21]. In the present case, the small lesion in the left kidney may have already exhibited structural fragility prior to surgery. We hypothesize that the ensuing systemic stress could have acted as a potential trigger for rupture and hemorrhage. However, we acknowledge that this remains a hypothesis, and causality cannot be definitively established based on a single case. These observations raise the question of whether, in patients with suspicious contralateral renal lesions identified preoperatively, perioperative strategies such as strict blood pressure control, avoidance of intense coughing or increased intra-abdominal pressure might have preventive value. Further studies are needed to investigate this possibility.

Thirdly, the rapid deterioration in this patient’s condition was marked by a series of severe and interrelated multisystem complications, with complex pathophysiological mechanisms and highly challenging clinical management. The initial postoperative presentation of fever, cough, and elevated inflammatory markers suggested a possible postoperative infection or inflammatory response. This was soon followed by spontaneous rupture of the left kidney with massive hemorrhage, leading to acute blood loss and hypovolemic shock. This event caused a profound insult to the patient’s circulatory system and may have further exacerbated systemic inflammatory response syndrome (SIRS) through mechanisms such as ischemia-reperfusion injury and transfusion-related immunomodulation (TRIM) [22]. This intense systemic inflammatory state served as the key pathophysiological basis for the development of ARDS. At the same time, surgical trauma, hemorrhagic stress, and subsequent ICU interventions—such as mechanical ventilation—significantly impaired the patient’s immune defenses [23–25], creating conditions conducive to secondary mixed infections. In this case, co-infection with influenza A and B viruses, bacteria, and urinary fungal infection was identified. The development of chylothorax in our patient may be explained by several etiological hypotheses. First, subtle mechanical injury to the thoracic duct or minor lymphatic branches during surgery or central venous catheter placement could have resulted in chyle leakage, even if undetectable by bedside ultrasound [26]. Second, severe infection or systemic inflammation may increase lymphatic permeability, promoting chyle extravasation into the pleural space [27]. Third, functional or structural abnormalities of the lymphatic system, possibly exacerbated by multiorgan dysfunction or hemodynamic fluctuations, may have contributed to the formation of chylothorax [28, 29]. Fourth, elevated central venous pressure (CVP) secondary to aggressive fluid resuscitation for hemorrhagic shock and ARDS is a well-documented cause of non-traumatic chylothorax in critically ill patients [30]; however, in this patient, CVP levels were not markedly elevated during the ICU course (peak CVP 11 mmHg), arguing against this as the primary mechanism. While the exact mechanism remains uncertain, considering these hypotheses provides a framework for understanding the observed chylothorax and guiding supportive management. These complications interacted with one another, forming a vicious cycle of “hemorrhage → shock → inflammation/ARDS → immunosuppression → infection → nutritional and metabolic disturbances,” which greatly increased the complexity of treatment and the difficulty of clinical management.

It is worth emphasizing that the left renal lesion in this case did contain macroscopic fat visible on non-contrast CT, which should have raised suspicion for angiomyolipoma. However, its atypical patchy appearance and the lack of contrast-enhanced imaging prevented a definitive preoperative diagnosis. This experience underscores that even non-contrast CT findings of focal fat density in the contralateral kidney warrant further characterization with contrast-enhanced CT or fat-suppressed MRI prior to elective urological surgery, regardless of the patient’s asymptomatic status.

In the management of spontaneous renal rupture, treatment options include conservative management, selective arterial embolization (SAE), and surgical intervention. Conservative management—comprising bed rest, fluid resuscitation, blood transfusion, and blood pressure control—is appropriate for hemodynamically stable patients with limited bleeding [2]. Surgical intervention, including partial or total nephrectomy, is reserved for patients with hemodynamic instability despite resuscitation or those with failed embolization [31]. According to current literature, SAE is the preferred treatment for hemodynamically stable patients due to its minimally invasive nature and ability to preserve renal parenchyma [2, 31]. In the present case, the patient presented with massive hemorrhage and hemodynamic fluctuations. SAE was therefore selected as it achieved rapid hemostasis while minimizing surgical trauma, which aligns with the treatment strategy recommended in the literature [2, 31].

Finally, the successful management of this case eloquently demonstrates the central value of MDT collaboration in treating complex and critically ill patients. From the timely intervention by urology and interventional radiology in managing the renal rupture and hemorrhage—achieving rapid hemostasis via arterial embolization—to the intensive care unit’s leadership in providing mechanical ventilation for ARDS and maintaining vital functions, each stage of care was essential. This was complemented by the infectious diseases team’s targeted antimicrobial therapy against mixed pathogens, and the thoracic surgery and nutrition teams’ expert management of chylothorax through drainage and tailored nutritional support. Every critical decision and therapeutic step relied on close collaboration and seamless coordination among the involved specialties. The MDT approach ensured a treatment plan that was comprehensive, timely, and individualized, forming the organizational foundation that ultimately broke the vicious cycle and enabled successful recovery. This case highlights the importance of establishing a routine MDT mechanism for the management of urological emergencies complicated by severe systemic conditions.

## Conclusion

This rare case serves as a powerful reminder: preoperative evaluation in urological surgery must consider both kidneys, with vigilance for subtle or indeterminate contralateral renal abnormalities; during the perioperative period, attention should be paid to the potential impact of systemic stress responses on vulnerable target organs; in the face of complex, multisystem complications, a deep understanding of their underlying pathophysiological connections is essential. Throughout the course of management, multidisciplinary collaboration remains the most effective tool in navigating such critical clinical challenges and saving lives. This case report aims to enhance clinicians’ awareness of such complex scenarios and to help optimize diagnostic and therapeutic strategies.

## Supplementary Information

Supplementary Material 1.

## Data Availability

All relevant data supporting the conclusions of this article are included within the manuscript.

## References

[1] Mariolis-Sapsakos T, Nannou E, Angelis S, Filippou D. Wunderlich Syndrome: Spontaneous Cystic Rupture on Account of Acquired Kidney Atrophy. Cureus. 2022;14(10):e30386. 10.7759/cureus.30386. 36407245 10.7759/cureus.30386 PMC9668205

[2] Shah JN, Gandhi D, Prasad SR, Sandhu PK, Banker H, Molina R, Khan S, Garg T, Katabathina VS. Wunderlich Syndrome: Comprehensive Review of Diagnosis and Management. Radiographics. 2023;43(6):e220172. 10.1148/rg.220172. 37227946 10.1148/rg.220172

[3] Giovini M, Poggiali E, Zocchi P, Bianchi E, Antonucci E, Barbera M. A Case of Spontaneous Renal Haemorrhage (Wunderlich Syndrome) in an Anticoagulated Patient. Eur J Case Rep Intern Med. 2022;9(4):003269. 10.12890/2022_003269. 35520370 10.12890/2022_003269 PMC9067415

[4] Kim JW, Kim JY, Ahn ST, Park TY, Oh MM, Moon DG, Park HS. Spontaneous perirenal hemorrhage (Wunderlich syndrome): An analysis of 28 cases. Am J Emerg Med. 2019;37(1):45–7. 10.1016/j.ajem.2018.04.045. 29779678 10.1016/j.ajem.2018.04.045

[5] Larbi H, Hassan I, Cherraqi A, Saouab R, Mohammed A, Ahmed A. Wunderlich syndrome: Rare and unrecognized emergency. Urol Case Rep. 2022;43:102093. 10.1016/j.eucr.2022.102093. 35520030 10.1016/j.eucr.2022.102093 PMC9065635

[6] Zhang JQ, Fielding JR, Zou KH. Etiology of spontaneous perirenal hemorrhage: a meta-analysis. J Urol. 2002;167(4):1593–6. 10.1097/00005392-200204000-00006. 11912370 10.1097/00005392-200204000-00006

[7] Desborough JP. The stress response to trauma and surgery. Br J Anaesth. 2000;85(1):109 – 17. 10.1093/bja/85.1.109. PMID: 10927999. 10.1093/bja/85.1.109 10927999

[8] Kohl BA, Deutschman CS. The inflammatory response to surgery and trauma. Curr Opin Crit Care. 2006;12(4):325–32. 10.1097/01.ccx.0000235210.85073.fc. 16810043 10.1097/01.ccx.0000235210.85073.fc

[9] Kehlet H. Multimodal approach to control postoperative pathophysiology and rehabilitation. Br J Anaesth. 1997;78(5):606–17. 10.1093/bja/78.5.606. 9175983 10.1093/bja/78.5.606

[10] Gagnier JJ, Kienle G, Altman DG, Moher D, Sox H, Riley D, et al. The CARE Guidelines: Consensus-based Clinical Case Reporting Guideline Development. Glob Adv Health Med. 2013;2(5):38–43. 10.7453/gahmj.2013.008. 24416692 10.7453/gahmj.2013.008 PMC3833570

[11] Panthongviriyakul C, Bines JE. Post-operative chylothorax in children: an evidence-based management algorithm. J Paediatr Child Health. 2008;44(12):716–21. 10.1111/j.1440-1754.2008.01412.x. 19077067 10.1111/j.1440-1754.2008.01412.x

[12] Waikar HD, Kamalaneson P, Mohamad Zamri MS, Jayakrishnan AG. Chylothorax after off-pump coronary artery bypass graft surgery: Management strategy. Ann Card Anaesth. 2018;21(3):300–3. 30052221 10.4103/aca.ACA_212_17 PMC6078034

[13] Zheng J, Chen YY, Zhang CY, Zhang WQ, Rao ZY. The retrospective research of enteral nutrition with medium-chain triglyceride and total parenteral nutrition support of postoperative chylothorax in adults. SAGE Open Med. 2020;8:2050312120938221. 10.1177/2050312120938221. 32655864 10.1177/2050312120938221 PMC7331756

[14] Hindman N, Ngo L, Genega EM, Melamed J, Wei J, Braza JM, Rofsky NM, Pedrosa I. Angiomyolipoma with minimal fat: can it be differentiated from clear cell renal cell carcinoma by using standard MR. techniques? Radiol. 2012;265(2):468–77. 10.1148/radiol.12112087. 10.1148/radiol.12112087 PMC3480813 23012463

[15] Thiravit S, Teerasamit W, Thiravit P. The different faces of renal angiomyolipomas on radiologic imaging: a pictorial review. Br J Radiol. 2018;91(1084):20170533. 10.1259/bjr.20170533. 29327940 10.1259/bjr.20170533 PMC5965995

[16] Thapa N, Maharjan S, Hona A, Pandey J, Karki S. Spontaneous rupture of renal angiomyolipoma and its management: A case report. Ann Med Surg (Lond). 2022;79:104037. 10.1016/j.amsu.2022.104037. 35860139 10.1016/j.amsu.2022.104037 PMC9289389

[17] Reysner T, Wieczorowska-Tobis K, Kowalski G, Grochowicka M, Pyszczorska M, Mularski A, Reysner M. The Influence of Regional Anesthesia on the Systemic Stress Response. Rep (MDPI). 2024;7(4):89. 10.3390/reports7040089. 10.3390/reports7040089 PMC12199975 40757696

[18] Reikeras O, Borgen P, Reseland JE, Lyngstadaas SP. Changes in serum cytokines in response to musculoskeletal surgical trauma. BMC Res Notes. 2014;7:128. 10.1186/1756-0500-7-128. 24602333 10.1186/1756-0500-7-128 PMC3975856

[19] Mishani S, Belhoul-Fakir H, Lagat C, Jansen S, Evans B, Lawrence-Brown M. Stress distribution in the walls of major arteries: implications for atherogenesis. Quant Imaging Med Surg. 2021;11(8):3494–505. 10.21037/qims-20-614. 34341726 10.21037/qims-20-614 PMC8245957

[20] Maucher D, Schmidt B, Schumann J. Loss of Endothelial Barrier Function in the Inflammatory Setting: Indication for a Cytokine-Mediated Post-Transcriptional Mechanism by Virtue of Upregulation of miRNAs miR-29a-3p, miR-29b-3p, and miR-155-5p. Cells. 2021;10(11):2843. 10.3390/cells10112843. 34831066 10.3390/cells10112843 PMC8616298

[21] Voirin AC, Perek N, Roche F. Inflammatory stress induced by a combination of cytokines (IL-6, IL-17, TNF-α) leads to a loss of integrity on bEnd.3 endothelial cells in vitro BBB model. Brain Res. 2020;1730:146647. 10.1016/j.brainres.2020.146647. 31911168 10.1016/j.brainres.2020.146647

[22] Vázquez-Galán YI, Guzmán-Silahua S, Trujillo-Rangel WÁ, Rodríguez-Lara SQ. Role of Ischemia/Reperfusion and Oxidative Stress in Shock State. Cells. 2025;14(11):808. 10.3390/cells14110808. 40497985 10.3390/cells14110808 PMC12154509

[23] Islam MN, Bradley BA, Ceredig R. Sterile post-traumatic immunosuppression. Clin Transl Immunol. 2016;5(4):e77. 10.1038/cti.2016.13. 10.1038/cti.2016.13 PMC4855263 27195120

[24] Brakenridge SC, Wang Z, Cox M, Raymond S, Hawkins R, Darden D, Ghita G, Brumback B, Cuschieri J, Maier RV, Moore FA, Mohr AM, Efron PA, Moldawer LL. Distinct immunologic endotypes are associated with clinical trajectory after severe blunt trauma and hemorrhagic shock. J Trauma Acute Care Surg. 2021;90(2):257–67. 10.1097/TA.0000000000003029. 33214489 10.1097/TA.0000000000003029 PMC8194286

[25] Keane S, Martin-Loeches I. Host-pathogen interaction during mechanical ventilation: systemic or compartmentalized response? Crit Care. 2019;23(Suppl 1):134. 10.1186/s13054-019-2410-0. 31200727 10.1186/s13054-019-2410-0 PMC6570626

[26] Saxena P, Shankar S, Kumar V, Naithani N. Bilateral chylothorax as a complication of internal jugular vein cannulation. Lung India. 2015;32(4):370–4. 10.4103/0970-2113.159579. 26180388 10.4103/0970-2113.159579 PMC4502203

[27] Agustin M, Yamamoto M, Tongma C, Chua LA, Torres M, Shay S. Spontaneous Chylothorax following Septic Pulmonary Embolization. Case Rep Pulmonol. 2020;2020:3979507. 10.1155/2020/3979507. 32148992 10.1155/2020/3979507 PMC7057015

[28] Bakar B, Pampal K, Tekkok IH. Infected bilateral chylothorax in a problematic case. J Curr Surg. 2012;2(2):62–5.

[29] Ur Rehman K, Sivakumar P. Non-traumatic chylothorax: diagnostic and therapeutic strategies. Breathe (Sheff). 2022;18(2):210163. 10.1183/20734735.0163-2021. 36337134 10.1183/20734735.0163-2021 PMC9584559

[30] Bhatnagar M, Fisher A, Ramsaroop S, Carter A, Pippard B. Chylothorax: pathophysiology, diagnosis, and management-a comprehensive review. J Thorac Dis. 2024;16(2):1645–61. 10.21037/jtd-23-1636. 38505027 10.21037/jtd-23-1636 PMC10944732

[31] Jo SB, Ahn ST, Oh MM, Park SJ, Yoon YH, Kim JW, et al. Analysis of 46 Cases of Spontaneous Perirenal Hemorrhage: A Retrospective Observational Study. J Clin Med. 2025;14(9):2986. 10.3390/jcm14092986. 40364017 10.3390/jcm14092986 PMC12072988

