# Supplementary material for: Postoperative contralateral renal rupture with multisystem complications after surgery for right renal calculi: a rare clinical case report
Source: BMC Surg. 2026 Apr 24;26:403. doi: 10.1186/s12893-026-03773-8 (PMC13262454; doi:10.1186/s12893-026-03773-8)
Supplement: Supplementary file 1 — Supplementary Material 1. [file 12893_2026_3773_MOESM1_ESM.docx]

**Supplementary Table 1.** Peak and nadir values of key laboratory parameters and daily chest drainage volume during hospitalization.

| Indicator (Unit) | Reference Range | Extremum Type | Extremum Value | Corresponding Hospitalization Day |
| --- | --- | --- | --- | --- |
| Hemoglobin (g/L) | 115–150 | Nadir | 58 | Day 5 |
| Oxygen Saturation (%) | ≥95 | Nadir | 70 | Day 6 |
| C-Reactive Protein (CRP, mg/L) | <5 | Peak | 91 | Day 16 |
| Albumin (g/L) | 40–55 | Nadir | 27 | Day 8 |
| Serum Creatinine (μmol/L) | 41–81 | Nadir | 40 | Day 14 / 25 / 30 |
| Platelet (×10⁹/L) | 100–300 | Peak | 433 | Day 17 |
| International Normalized Ratio (INR) | 0.8–1.24 | Peak | 1.1 | Day 6 |
| Activated Partial Thromboplastin Time (APTT, seconds) | 21–35 | Peak | 27.1 | Day 0 |
| Fibrinogen (g/L) | 2–4 | Peak | 4.27 | Day 6 |
| D-Dimer (g/L) | <0.55 | Peak | 7.52 | Day 6 |
| Procalcitonin (PCT, μg/L) | <0.5 | Peak | 0.728 | Day 11 |
| Chest Drainage Volume (mL) | - | Peak | 800 | Day 10 |
